# Exploring the Impact of Labour Mobility on the Mental Health and Wellbeing of Skilled Trades Workers in Ontario, Canada

**DOI:** 10.3390/ejihpe13080105

**Published:** 2023-08-05

**Authors:** Vijay Kumar Chattu, Ali Bani-Fatemi, Aaron Howe, Behdin Nowrouzi-Kia

**Affiliations:** 1 ReSTORE Lab, Department of Occupational Science and Occupational Therapy, Temerty Faculty of Medicine, University of Toronto, Toronto, ON M5G 1V7, Canada; a.bani.fatemi@mail.utoronto.ca (A.B.-F.); aa.howe@utoronto.ca (A.H.); 2 Center for Evidence-Based Strategies (CEBS), Global Health Research and Innovations Canada Inc. (GHRIC), 100 Consilium Place, Scarborough, ON M1H 3E3, Canada; 3 Center for Transdisciplinary Research, Saveetha Dental College and Hospitals, Saveetha Institute of Medical and Technical Sciences, Saveetha University, Chennai 600077, India; 4 Krembil Research Institute, University Health Network, 60 Leonard Ave., Toronto, ON M5T 0S8, Canada; 5 Centre for Research in Occupational Safety & Health, Laurentian University, 935 Ramsey Lake Rd., Sudbury, ON P3E 2C6, Canada

**Keywords:** mental health, labour mobility, skill trades workers, migration, supercommuting, burnout, job stress, Ontario, Canada

## Abstract

Labour mobility and subsequent workers migration is an increasing trend worldwide and can be a force that counteracts Canada’s shortage of skilled labour. Supercommuting allows workers facing economic challenges to pursue more financially advantageous work opportunities in other regions. This study aimed to evaluate the “supercommuting” labour mobility model and its impact on long-distance mobile workers’ mental health and wellbeing. We utilized a non-experimental research design using convenience sampling from workers who participated in Blue Branch Inc.’s (Hamilton, Canada) supercommuting labour mobility model. An online questionnaire collected demographic data, work-related data, occupational stress measures related to burnout, and job-related stress data. Data collection was started on 1 April 2021, and of the total 58 participants, the majority (44, 76%) were male, born outside Canada, and had an average age of 32.8 years. Workplace Safety (95%), full-time employment opportunity (95%), career advancement possibility (95%), and income and benefits (94.9%) were found to be the most crucial factors to keep study participants working in their current position. Of the 47 participants who experienced burnout, only one showed severe burnout in each domain (personal, work-related, and colleague-related). There is a great need for preventative burnout programs and supportive employer resources for those who engage in long-distance labour commuting. The study emphasizes the need to encourage policymakers to develop solutions for training future Ontario workers to support mobile employment and long-distance labour commuting.

## 1. Introduction

The COVID-19 pandemic has profoundly and drastically impacted the global labour market. The unprecedented pandemic led to dramatic unemployment rates for millions worldwide and a disproportionately negative impact on labour mobility. Many migrant workers engage in labour mobility and played a substantial role in working throughout the pandemic and providing essential services. Globally, international migrants were estimated to be about 281 million in 2020, making up 3.6% of the global population [1]. Almost two-thirds of global migrants were labour migrants, 48 percent were females, and 74 percent were working age 20–64 years [1,2]. Labour mobility is a complex process that requires movement between workplaces, occupations, and industries [3], which helps individuals find work where needed, with the possibility of higher wages and increased productivity when matched with jobs best suited for them. Labour mobility and subsequent workers migration is an increasing trend worldwide, with migrant workers making up a significant quota of economic growth in high-income countries. Labour mobility is affected by several socioeconomic and personal determinants, such as policies and economic conditions, age, gender, marital status, education, occupation, national origin, and health, respectively [3].

Employers may incur significant expense for relocation costs for their employees. In addition, the social exclusion of migrant workers can also further threaten workers’ sense of belonging post-migration from one work region to another [4]. Supercommuting is defined as short-term (typically 6-week rotations) labour mobility that allows workers to be transported from areas of high unemployment to employers looking for skilled workers across Canada [5]. It could be as short as 14 days on and 14 days off up to rotations that can last six weeks and one week off on a fly-in and fly-out (FIFO) basis (or what is referred to in the industry as FIFO). The skilled trade sectors in Canada (construction, manufacturing, services, and automotive) employ about 20% of Canadians [6]. There has been a decrease in the number of skilled trades workers over the years. In 2019, the annual number of newly certified tradespeople declined by 3.9% [7]. This decrease in skilled trades workers in the industry can also be seen in migrant populations; the proportion of populations migrating to other provinces for work has fallen from roughly 2% in the 1970s to around 1% in 2015 [8].

Additionally, Canada’s aging workforce also threatens the growth of the country’s labour force, with competitive workers becoming harder to retain in work environments as they age [9]. In Ontario, there is an ongoing shortage in labour supply, particularly within the skilled trades [10]. Between 2020 and 2022, the ratio of unemployed individuals to vacant positions in Ontario decreased from 2.6 to 1.5 [11]. The recruitment of skilled workers is expected to be a challenge for nearly 40% of the business, particularly in sectors that rely on skilled labour [12]. The field faces disparate structures for supporting apprenticeships, an aging population, and barriers to access opportunities for labour mobility.

Recurrent labour mobility is linked to mental health disorders, such as anxiety, depression, alcohol-related disorders, and other conditions, such as ischemic heart disease, duodenal ulcer, and stroke [3]. On the other hand, these conditions have shown an increased risk of subsequent labour mobility, thus inferring a bidirectional effect [3]. There is a paucity of data in the literature about labour mobility and its effect on mental health. Most available data focused on the negative effects of mental health and labour mobility, with few data reported on its positive effects [13]. Therefore, this study aimed to study labour mobility and its impact on mental health and wellbeing among workers in Ontario province. The specific objective was to evaluate the “supercommuting” labour mobility model’s impact on its long-distance mobile workers’ mental health. This research fills some of the gaps in our understanding of the effects of labour mobility and adds value to the existing knowledge base on the supercommunting model, the practical challenges faced by employees, and the overall impact on mental health and wellbeing.

## 2. Materials and Methods

### 2.1. Study Design

In this study, we utilized a cross-sectional research design using convenience sampling from workers who participated in Blue Branch Inc.’s (Hamilton, Canada) supercommuting labour mobility model. Convenience sampling was employed to understand the direct impacts of long-distance labour mobility on workers’ mental health.

### 2.2. Study Setting

Blue Branch Inc., an Ontario-based organization, was founded in 2016 as a social enterprise focused on labor mobility through collaborating with employers across the province, focusing on rural communities. Thus, Blue Branch demonstrates its commitment to assisting skilled tradesmen in their endeavors, assisting employers by providing high-quality workforce options, and assisting communities by cutting unemployment and encouraging economic stability. It employs solutions that swiftly and effectively link job seekers to access opportunities and services, e.g., the supercommuting model, which includes long-distance mobile workers and their physical and mental health impacts.

### 2.3. Study Participants

The inclusion criteria for the participants: (1) must be living in Ontario province, (2) aged 18–65, and (3) must be registered in the Blue Branch’s supercommunting model. The participants who are not residents of Ontario are ineligible and therefore excluded.

### 2.4. Data Collection

Data collection was started on 1 April 2021, and included a sample of 58 participants (all employees) after the consent. The participants were invited by email to participate in an online survey questionnaire, which was distributed by Blue Branch Inc. The survey questionnaire collected demographic data, work-related data, and occupational stress measures related to burnout and job-related stress. The survey was available for completion for 18 months. To prevent multiple participation of participants in the online survey, we clearly communicated the survey guidelines and instructions to the participants, emphasizing that the survey was intended for a single response per participant.

Furthermore, we assigned each participant a unique identifier (a participant ID) to help us identify and exclude duplicate entries. We also included validation measures within the survey, such as required fields or response limits, to ensure that participants provided valid and unique information. This helped us to identify and filter out duplicate or suspicious responses. Finally, we regularly monitored and reviewed the survey responses to identify any patterns or inconsistencies that might indicate multiple participations. In this regard, we looked for duplicate identifiers or similar response patterns to identify potential duplicates.

### 2.5. Study Instruments

The survey measures used in the study were created by the principal investigator, B.N.-K., and were based on validated questionnaires. Trained research staff administered the survey. The survey questionnaire consisted of 38 items and aimed to collect demographic data, including gender and sex, age, ethnicity, marital status, educational attainment, total years of work experience, daily and weekly work hours, overtime hours worked, income, and travel time required for work. Additionally, the questionnaire included an inventory of factors related to occupational stressors and burnout, which were assessed using the Copenhagen Burnout Inventory (CBI) [14] and the National Institute for Occupational Safety and Health Generic Job Stress Questionnaire (NIOSH Generic Job Stress Questionnaire) [15]. We used CBI to measure the level of burnout in the study participants. This questionnaire has three subdivisions covering personal burnout, work-related burnout, and client-related burnout. The level of physical and mental fatigue and exhaustion a person experiences is known as personal burnout. Work-related burnout is defined as the degree of physical and psychological exhaustion that a person considers to be related to their job. Client-related burnout is the level of physical and psychological exhaustion that a person considers to be related to their job with clients (colleagues in this study). To collect data and assess occupational stress, we also used the NIOSH Generic Job Stress Questionnaire created by the NIOSH, which is used globally. This standardized tool measures the critical contributors to occupational stress, such as physical environment, role ambiguity/conflict, level of control, administrative and co-worker support, workload, and skill demand. It includes concepts relating to stressors likely to cause occupational strain in the worker and elements that may influence how the worker responds to such stressors. As a result, the NIOSH Generic Work Stress Questionnaire was utilized to assess overall job stress using the domains outlined below: (i) job satisfaction, (ii) non-work activities, (iii) social support, (iv) workplace hazards, and (v) self-esteem. These items are measured on an index scale from 1 to 5.

### 2.6. Data Analysis

To account for missing data related to missing scales, or missing items within scales, we conducted multiple imputations by chained equations (MICE) using the MICE package in R version 3.16.0 [16]. The dataset containing all variables, including baseline and each follow-up point, was utilized, assuming missing at random (MAR). The data were analyzed in SPSS Statistics version 29 to determine the demographic and work-related predictors of stress and burnout using descriptive and inferential statistics. Appropriate checks were made to ensure that assumptions of normality were followed without any violations, apart from the application of quality assurance checks by re-entering 25% of the randomly selected sample. Weighted Cohen’s Kappa statistics were used to determine inter- and intra-examiner variability. The collected data were presented, summarized, and analyzed using various statistical methods such as graphs and/or diagrams and tables of central tendency (mean, median and mode), dispersion (range, standard deviation), etc.

## 3. Results

### 3.1. Demographic Characteristics of Study Participants

We achieved the target sample (n = 58) of employees, and the majority (n = 44, 76%) of the participants were male, born outside Canada, with an average age of 32.8 years as shown in Table 1. The findings showed that the majority of the participants described their ethnicity as Middle Eastern (22, 38%), followed by White North American (n = 9, 16%), and showed English as their primary language (n = 25, 43%). Most of the participants completed high school (19, 33%), followed by graduates with a university degree (n = 17, 30%). Regarding lifestyle factors, such as smoking, most of them were non-smokers. The participants included various sectors, such as electrical, welding, carpentry, construction and others.

### 3.2. Importance of Work-Related Factors

**Figure 1:**
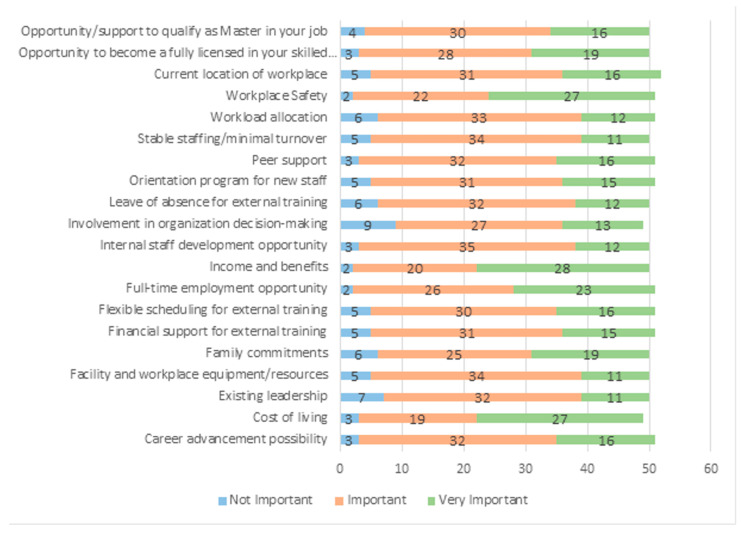
Factors that keep participants working in their current position at their workplace.

To find out the importance of the factors that keep employees working in their current position in their current workplace, we surveyed 19 work-related factors that we adopted from a previous questionnaire developed by Nowrouzi-Kia [14]. Workplace Safety (95%), full-time employment opportunity (95%), career advancement possibility (95%), and income and benefits (94.9%) were found to be the most crucial factors to keep study participants working in their current position at their current workplace (Figure 1). 

### 3.3. Availability and Satisfaction with the Work-Related Factors in the Current Workplace

**Figure 2:**
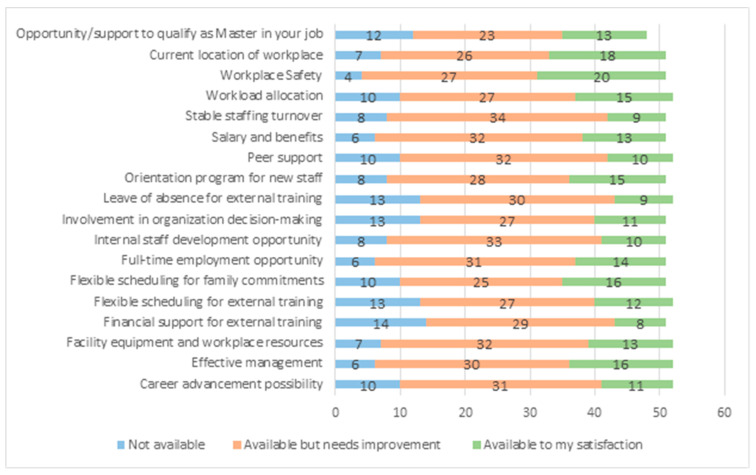
Availability and satisfaction with the following factors in the current workplace.

Similar to the previous questionnaire, the survey participants were assessed by an 18-factor self-reported questionnaire adopted from another questionnaire created by Nowrouzi-Kia in a previous study in order to identify the availability and satisfaction with those factors in their current workplace [17]. Workplace safety (40.5%), the current location of the workplace (38.1%), and flexible scheduling for family commitment and effective management (33.3%) were the most common factors that participants believed are available for their satisfaction in their current workplace (Figure 2).

### 3.4. Burnout

The level of burnout in the study participants that were analyzed using the CBI is shown in Table 2. Our findings showed that only one participant showed severe personal burnout (average score of 100). Of the few (n = 6) participants who experienced high burnout, one had a high score (average score of 75 to 99). In the work-related and colleague-related burnout categories, only two had experienced high burnout, of which one had a high score. Forty-five participants experienced moderate burnout (average score of 50 to 74), including personal burnout (n = 15), work-related burnout (n = 19), and colleague-related burnout (n = 11). Only one participant did not experience any personal, work-related, or colleague-related burnout.

We conducted non-parametric tests to explore potential associations between burnout characteristics (Personal Burnout, Work-related Burnout, and Colleague-related Burnout) and various factors (Age, Gender, Level of Education, Born in Canada, Trained in Ontario, and Employment Status). The findings suggest no evidence for an association between the burnout characteristic and the factors (Table 3).

## 4. Discussion

Labour mobility can be a force that counteracts Canada’s shortage in skilled labour as it allows workers who are facing economic conditions in their current workplace to pursue more financially advantageous work opportunities in other regions. However, there remain economic challenges to labour mobility. Our study investigated burnout and work-related satisfaction of mobile and long-distance labour commuting employees. We anticipated that there would be a higher proportion of burnout and work-related stress in this sample due to the long-term physical and mental health effects of long-distance labour commuting documented in the literature [3,18]. Our findings supported this hypothesis as we observed a high proportion of participants that were experiencing some form of moderate to severe burnout. However, we did not anticipate that the most common form of burnout participants would report personal burnout.

A Swedish study by Liljegren et al. found increased rates of mental health symptoms and burnout following mobility [19]. A similar study found that migrant workers had a greater prevalence of depression than non-migrant workers, with remarkably poor mental health among participants > 45 years of age and alcohol-related disorders were associated with identified work-related stress [20,21]. Causative factors of depression include working long hours, an unhealthy work environment, poor job satisfaction, social isolation, and poor physical health [20]. A similar study found that isolation was associated with anxiety, while enormous amounts of stress and high job demands were associated with depression [20]. Poor standard of living, social disparities, bias, and mobile status resulted in more depression and suicidal ideations [20].

Personal burnout is a state of prolonged stress and exhaustion related to an individual’s perception of their personal experience [14]. Personal burnout has been associated with work-related stress and has been shown to exacerbate it in other representative working samples [22,23]. The elevated levels of personal burnout observed in our sample may be due to several reasons, including a lack of available workplace resources [24], pre-existing personal emotional and social resources [25], or workplace exhaustion that depletes available resources for personal social roles [26]. We did not perform any analyses to measure these characteristics. Compared to other studies, we also found a similar prevalence of severe burnout (2.5%) relative to different working samples [27]. This suggests that mobile and long-distance labour commuting may not be associated with elevated levels of burnout relative to those living closer to their employment.

For those involved in the construction trades, workplace-related stress can occur at their employer’s workplace and job sites where they perform duties [18]. Therefore, the experience of stress can be unique among skilled trades workers as they experience external pressures from different workplace cultures, settings, and social systems [18]. Construction trades workers also experience stress through difficult physical working conditions, long hours (e.g., disruption of work-life balance), and physical and mental exhaustion [18]. There is a critical need for preventative measures to address burnout in this sample of long-distance commuting workers. Employers can use a few strategies of burnout intervention to reduce work-related stress based on our findings on the importance of work-related factors. Workplace safety, growth, and stability are employment characteristics that were most important to workers who completed the study; however, many participants did not intend to remain in their current position for the next 5 years. This juxtaposition is essential to evaluate because it appears to suggest that workers are devalued, and their experience of work-related burnout is driving them to consider different forms of employment. A recent study has shown that intervention programs related to workplace safety and personal efficacy (e.g., growth and resource development) through principles of rational emotive theory have been effective [27].

A few more studies highlight the psychological distress among these supercommuning groups. An Australian study among fly-in and fly-out (FIFO) workers by Parker et al. highlighted the risk of poor mental health among these FIFO workers and their partners. The study further identified that identifying short/even time rosters and providing permanent accommodation on-site and other social activities are beneficial for mental health [28]. Similarly, another study from Southern Australia by Bowers et al. among construction and mining workers has concluded that psychological distress is significantly higher among the remote workforce than in the overall Australian population [29]. Both of these studies from Western and Southern Australia support the findings of a Canadian study from Alberta province. In this study, Dorrow and colleagues highlighted significant mental health challenges, including a work culture of mistrust regarding employer commitment and support for mental health and wellbeing. The study further recommended a flexible rotational schedule, improvements in worksite conditions, and availability of mental health training and resources [30]. Thus, our study findings align with these studies, which explore labour mobility and its impact on the mental health and wellbeing of this supercommuting workforce.

### Limitations of the Study

There are a few limitations to this research study. First, our sample size is small and the demographics of our sample are diverse, which may not be generalizable to all the skilled trades (electrical, welding, carpentry, construction and others) involved in this study or specific to the construction industry. The employees experience work-related stress by interacting with available personal and workplace resources. Therefore, these different social system interactions may have complicated our findings. Second, we used convenience sampling to recruit participants from employees and employers in partnership with Blue Branch. This approach may bias our findings as those that engage in work-related stress and burnout research may be at the two extremes and may not represent Ontario’s long-distance labour commuting demographic. Third, the scale used to quantitatively measure burnout does not distinguish between physical and psychological fatigue. Therefore, we cannot ascertain which aspects of their current job perpetuate their experience of workplace stress and burnout. Lastly, our findings are cross-sectional and only represent a single time point at the time of survey completion. Future studies should examine the trajectory of burnout over time from a naturalistic perspective but also through the administration of a burnout intervention program.

## 5. Conclusions

This study concludes that labour mobility and the supercommunting workforce face psychological distress and experience poor mental health. In our study, most of the participants were males, mostly from the Middle East born outside Canada. The participants belonged to various industry sectors, such as electrical, welding, carpentry, construction, and others. The CBI used to measure burnout showed that a majority (45) of the participants experienced moderate burnout. Safety in the workplace, the current location of the workplace, the availability of flexible scheduling to accommodate family obligations, and effective management were the most frequently cited factors that participants believe contribute to their job satisfaction. The study emphasizes the need for preventative burnout programs and supportive employer resources for those who engage in long-distance labour commuting. The study highlights the need for developing solutions for training future Ontario workers to support mobile employment and long-distance labour commuting. Secondly, we recommend evaluating the effectiveness of Blue Branch’s “supercommuting” program to determine if there are any gaps or barriers in physical and mental support systems for those workers engaged in the program. There is a great need to analyze the obtained data to determine whether demographic or social factors influence engagement in the “supercommuting” program.

Given the criticality of studying this labour mobility in Ontario, we propose an expansion of this study to cover the whole Ontario province with 20 employers and 200 employees to evaluate influential factors associated with Ontario workers’ mental health status and access to mental health support, which we will achieve through interviews completed by the workers and employers to gain their perspectives. In addition, we believe this is the first study from the Canadian province of Ontario to examine this supercommuting model as a component of occupational health and safety, which has become increasingly prevalent since the pandemic. Further studies in this labour mobility domain should examine whether there is a predictive role of personal and workplace resources in the experience of burnout in those who engage in long-distance labour commuting.

## Figures and Tables

**Table 1:** Demographic characteristics of study participants (n = 58).

Variable		Employees (n = 58)
**Age (Years), Mean (SD)**		32.80 (12.70)
Gender, n (%)	Male	44 (75.9)
	Female	12 (20.7)
Ontario born, n (%)	Yes	6 (10.3)
	No	52 (89.7)
Canada born, n (%)	Yes	15 (25.9)
	No	43 (74.1)
Marital status, n (%)	Single	38 (65.5)
	Married	13 (22.4)
	Divorced	6 (10.3)
	Widowed	1(1.7)
Highest education level, n (%)	Incomplete high school	10 (17.2)
	Completed high school	19 (32.8)
	College certificate	3 (5.2)
	College diploma	6 (10.3)
	University graduate degree	17 (29.3)
	University undergraduate degree	1 (1.7)
Training in Ontario, n (%)	Yes	18 (31)
	No	37 (63.8)
Primary language, n (%)	English	25 (43.1)
	Persian	2 (3.4)
	Spanish	1 (1.7)
	Ukrainian	1 (1.7)
	Arabic	23 (39.7)
	Kurdish	1 (1.7)
	Turkish	1 (1.7)
	Dari	1 (1.7)
Ethnicity, n (%)	White European/North American	9 (15.5)
	Middle Eastern	22 (37.9)
	Asian East	2 (3.4)
	Mixed Background	1 (1.7)
	Other	6 (10.3)
	Prefer not to answer	3 (5.2)
	Black African	3 (5.2)
	Asian South	5 (8.6)
	Asian Southeast	1 (1.7)
	Black Caribbean	3 (5.2)
Aboriginal/Metis/Inuit, n (%)	Yes	1 (1.7)
	No	57 (98.3)
Employment status, n (%)	Full-time, contract	24 (41.4)
	Full-time, permanent	18 (31)
	Part-time, contract	4 (6.9)
	Part-time, permanent	6 (10.3)
	Other	4 (6.9)
Current smoker, n (%)	Yes	19 (32.8)
	No	37 (63.8)
Union, n (%)	Yes	2 (3.4)
	No	53 (91.4)
Stay in the current position for the next 5 years, n (%)		Yes
		No

**Table 2:** Copenhagen Burnout Inventory (n = 45).

Type of Burnout	Mean [SD]	Minimum Score	Maximum Score	Median	Moderate Burnout * (n)	High Burnout * (n)	Severe Burnout * (n)
Personal burnout	46.41 [22.09]	0.00	100	45.83	15	6	1
Work-related burnout	43.21 [19.03]	14.28	100	42.85	19	2	1
Colleague-related burnout	30.36 [22.94]	0.00	100	25.00	11	2	1

* In the Copenhagen Burnout Inventory, scores of 50 to 74 are deemed to be ‘moderate burnout’, 75–99 is deemed to be high burnout and a score of 100 is deemed to be severe burnout.

**Table 3:** Association between burnout and the other variables.

Type of Burnout	Variable	*p*-Value
Personal Burnout	Age	0.78
	Gender	0.80
	Level of Education	0.59
	Born in Canada	0.20
	Trained in Ontario	0.85
	Employment Status	0.79
Work-related Burnout	Age	0.26
	Gender	0.36
	Level of Education	0.83
	Born in Canada	0.31
	Trained in Ontario	0.44
	Employment Status	0.68
Colleague-Related Burnout	Age	0.25
	Gender	0.60
	Level of Education	0.53
	Born in Canada	0.97
	Trained in Ontario	0.85
	Employment Status	0.90

## Data Availability

Data can be provided upon request from the corresponding author B.N.-K.
